# Rapid ecological isolation and intermediate genetic divergence in lacustrine cyclic parthenogens

**DOI:** 10.1186/1471-2148-10-166

**Published:** 2010-06-05

**Authors:** Katie S Costanzo, Derek J Taylor

**Affiliations:** 1 Department of Biological Sciences, The State University of New York at Buffalo, Buffalo, NY, 14260, USA; 2 Illinois Natural History Survey, Institute of Natural Resource Sustainability University of Illinois at Urbana-Champaign, IL 61802, USA

## Abstract

**Background:**

Ecological shifts can promote rapid divergence and speciation. However, the role of ecological speciation in animals that reproduce predominantly asexually with periodic sex and strong dispersal, such as lacustrine cladocerans, is poorly understood. These life history traits may slow or prevent ecological lineage formation among populations. Proponents of the postglacial ecological isolation hypothesis for *Daphnia *suggest that some species have formed postglacially in adjacent, but ecologically different habitats. We tested this hypothesis with ecological, morphological, and multilocus coalescence analyses in the putative lacustrine sister species, *Daphnia parvula *and *Daphnia retrocurva*.

**Results:**

*Daphnia parvula *and *D. retrocurva *showed strong habitat separation with rare co-occurrence. Lakes inhabited by *D. parvula *were smaller in size and contained lower densities of invertebrate predators compared to lakes containing *D. retrocurva*. In the laboratory, *D. retrocurva *was less vulnerable to invertebrate predation, whereas *D. parvula *was less vulnerable to vertebrate predation and was smaller and more transparent than *D. retrocurva*. The species are significantly differentiated at mitochondrial and nuclear loci and form an intermediate genetic divergence pattern between panmixia and reciprocal monophyly. Coalescence and population genetic modelling indicate a Late or Post Glacial time of divergence with a demographic expansion.

**Conclusions:**

Despite their young age and mixed breeding system, *D. parvula *and *D. retrocurva *exhibit significant ecological and genetic divergence that is coincident with the formation of deep temperate glacial lakes. We propose that predation may have facilitated the rapid divergence between *D. parvula *and *D. retrocurva *and that intermediate divergence of aquatic cyclic parthenogens is likely more common than previously thought.

## Background

Divergent selection for different habitats has long been proposed as a contributing factor to the evolution of species [1-4]. Regardless of geographic associations, ecological speciation from strong single selective agents, such as predation, or from the additive effects of multiple selective agents (i.e., the niche dimensionality hypothesis) now have empirical support [5-10]. A remaining theoretical gap is a detailed understanding of the role of the breeding system in speciation [11-14]. Although many eukaryotic taxa reproduce both sexually and asexually [13], there are still few empirical studies of speciation beyond strictly sexual taxa [14-17]. Because many organisms with mixed breeding systems are excellent dispersers [18-21], breeding systems with even a small amount of sexual reproduction should suffer homogenization of ecologically differentiated lineages [11,12,15,22]. Thus, taxa with mixed breeding systems coupled with high dispersal should possess a weak capacity for rapid ecological radiation compared to strict asexuals and sexuals [11,23-26]. Others, however, have proposed that priority effects where early colonization offers an advantage and divergent selection are frequently strong enough to overcome the homogenizing effects of such mixed breeding systems [22-27].

Cladocerans contain several candidate groups for detailed empirical study of ecological and genetic divergence in a system characterized by both sexual and asexual reproduction (cyclic parthenogenesis). These microcrustaceans often inhabit insular freshwater systems (i.e. lakes, ponds, reservoirs) with a mosaic of selective regimes through many environmental factors such as ephemerality, temperature, nutrient availability, competition, and predation [28-30]. For example, a common contrast occurs between lakes with visual vertebrate predators that can select for smaller prey species, and lakes dominated by gape-limited invertebrate predators that favor cladoceran communities dominated by large or helmeted species [31-36], as prey vulnerability is a function of prey size relative to predator size. Predation has been proposed to drive evolutionary divergence in morphology and behaviour [32,37-41] and speciation in cladocerans [23,25,26,41-43]. Still, there is little knowledge of ecological speciation among sister species of lacustrine cladocerans, where sexual reproduction is relatively infrequent.

**Figure 1:**
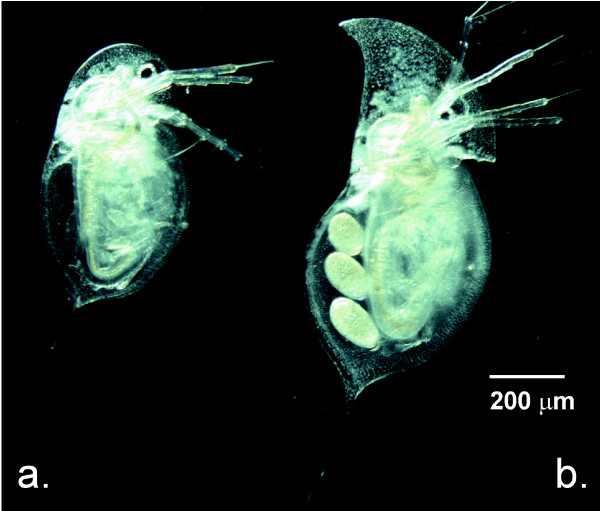
**(a.) *Daphnia parvula *adult female, (b.) *Daphnia retrocurva *adult gravid female**.

**Figure 2:**
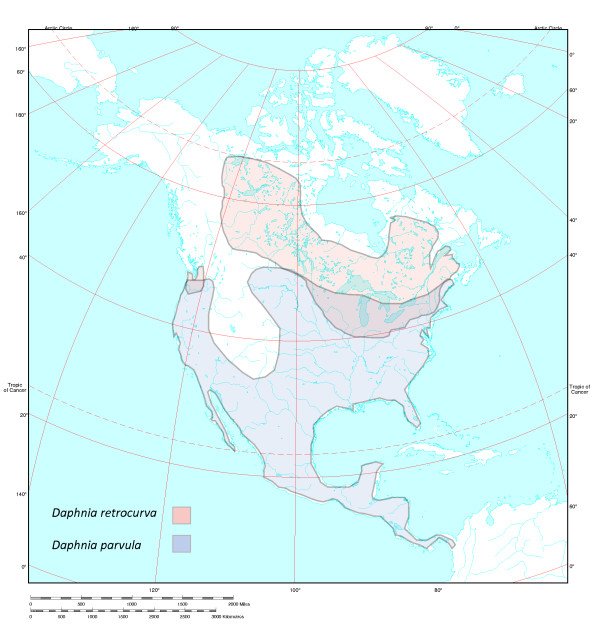
**Geographic distributions of *Daphnia parvula *and *Daphnia retrocurva *in North America**. The ranges show a large zone of overlap near the southern Great Lakes and are derived from Brooks [42].

The cladocerans, *Daphnia parvula *Fordyce, 1901 and *Daphnia retrocurva *Forbes, 1882 (Fig. 1), provide an opportunity to study the role of ecological factors in promoting rapid divergence in potentially young lacustrine taxa with a mixed breeding system. Although there is variation in the frequency of sex among populations, both species typically reproduce predominantly by clonal asexual reproduction with a few periodic episodes of sex throughout the season [42,44-47]. In North America, *D*. *retrocurva *has a more northerly distribution occurring from the central midwest through northern Canada, whereas *D*. *parvula *occurs from South America through the southern parts of Canada. However, the species overlap largely near the southern extent of the glacial lake zone in North America where rare co-occurrence is documented (Fig. 2), [42,48-50]. Brooks [42] proposed that more northerly *D. retrocurva *is a postglacial derivative of *D. parvula *based on their distribution and *D. retrocurva *inhabiting more novel post-glacial large lake habitats. The two species have a sister group relationship, but no multi-population genetic studies or detailed studies of genetic divergence have been carried out [51-54].

*Daphnia parvula *lacks the anterior retrocurved helmet (Fig. 1) that appears to be controlled by polyploid cells in the head region of *D. retrocurva *[55]. The helmet undergoes cyclomorphosis in *D. retrocurva *[32,56-60], which can be enhanced by cues from invertebrate predators [32,58-60] and laboratory studies also show decreased vulnerabilities of *D. retrocurva *to invertebrate predation with increased helmet size [61]. Importantly, multigenerational lab culture experiments failed to convert the head shape of *D. retrocurva *into the non-helmeted head shape of *D. parvula *[56,62,63]. Although the body size and tail spine length in *D. parvula *can be influenced by predator cues, attempts to induce helmet production have also been unsuccessful [59], suggesting the morphological differences between these two species likely has a genetic component. Additionally, although *D. retrocurva *is a classic example of seasonal polymorphism, experiments reveal that phenotypic plasticity also fails to explain the morphological differences between the two species [62,63].

Although there are no studies that specifically address ecological differentiation between *D. retrocurva *and *D. parvula*, there is substantial evidence that the interaction of predation with helmet phenotypes is evolutionarily and ecologically relevant. Kerfoot and Weider [63] for example, show that the abundance of *D. retrocurva *and larger size classes of helmets in *D. retrocurva*, are positively associated with the increased abundance and size of the invertebrate cladoceran predator, *Leptodora*, and reduced levels of planktivorous fish over time. Likewise, following the introduction of *Leptodora *in a lake, a shift from *D. parvula *(without the helmet) was observed to helmeted or large *Daphnia *[35], suggesting a role for predation as a selective agent for helmets. In contrast, a sixteen-year study [64] found repeated smooth, continuous transitions between large bodied *Daphnia *and small *Daphnia *(including *D. parvula*) associated with planktivorous fish density. Additionally, the abundance of *D. retrocurva *in glacial lakes is positively correlated with the abundance of invertebrate predators, and the abundance of introduced *D. parvula *is negatively associated with rates of invertebrate predation during a season [47]. Taken together, the translocation and natural experiments are consistent with divergent selection from predation playing an isolating role, which could also affect genetic differentiation. However, Lukaszewski et al. [65] found evidence that ecological conditions beyond predation can prevent the successful colonization of *D. retrocurva *into a lake containing *D. parvula*.

Isolation affects population genetic patterns in a time dependent fashion. Omland et al. [66] defined "intermediate divergence" as the stages between panmixis and reciprocal monophyly. The lineage divergence continuum, as visualized by networks, begins with gene frequencies diverging with only older internal haplotypes shared (less frequent haplotypes arising since isolation are private), followed by a lack of haplotype sharing. Eventually, population-specific clades (monophyly or paraphyly) are formed. In post-glacial isolation, which has been proposed by Brooks for *D. parvula *and *D. retrocurva *[42], such intermediate divergence is often produced and haplotype sharing is limited to the older central haplotypes of the networks [67-69]. In contrast, if isolation is caused by multiple glacial refugia during the late Pleistocene, patterns of multiple geographic subclades along with multiple demographic expansions is predicted as observed in other *Daphnia *[67,68].

Here, we specifically test for ecological and genetic divergence between *D. parvula *and *D. retrocurva*. Ecological speciation predicts that populations of *D. parvula *and *D. retrocurva *whose ranges overlap will be associated with ecologically different habitats. If predation is a major selective agent, then we should detect differences in the relative vulnerabilities to habitat-specific predators. If the morphotypes of "*retrocurva*" and "*parvula*" are isolated, then we expect to detect significant genetic differentiation in the zone of overlap. Further, under the postglacial isolation hypothesis, we expect intermediate divergence (lack of monophyly), a lack of multiple demographic expansions, and divergence times from coalescence modelling to be Late or Post-Glacial. We test these objectives through data culled from pre-existing databases, laboratory predation experiments, and population genetic analyses.

## Results

### Habitat differentiation

Of the 64 lakes analyzed from the data collected from the Environmental Protection Agency (EPA) of lakes in the Northeastern United States, 47 contained *D. parvula*, 16 contained *D. retrocurva*, and one contained both species. The Discriminant Analysis (DA) indicated that abiotic variables significantly discriminated between lakes containing *D. parvula *and lakes containing *D. retrocurva *(Table 1), with lake area, lake depth, and lake volume contributing most to this group separation. The Analysis of Variance (ANOVA) for lake volume was significant (F _1, 62 _= 7.36, P = 0.0079), with *D. retrocurva *inhabiting lakes of larger volume than those of *D. parvula *(400.35 × 10^5 ^m^3 ^± 34 × 10^5^, 41.34 × 10^5 ^m^3 ^± 16 × 10^4^, mean ± 1 standard error, respectively).

**Table 1:** Discriminant Analyses on abiotic variables.

Variable	Canonical variate correlation coefficients
Calculated Alkalinity (ueq/L)	-0.119
Chlorophyll A (ug/L)	0.306
Ionic Strength (M)	0.093
Total Nitrogen (ug/L)	0.273
Total Phosphorus (ug/L)	0.368
Mean Secchi dish depth (m)	-0.166
pH	-0.149
Total Suspended Solids (ug/L)	0.220
Turbulence (NTU)	0.230
**Mean Lake Depth (m)**	**-0.551**
**Lake Volume (m^3^)**	**-0.562**
**Lake Area (ha)**	**-0.493**
Lake Elevation	-0.197
% Watershed in human disturbed land	0.273

Wilks' Lambda *P*	0.007

Canonical variate correlation coefficients derived from the discriminant analysis for each abiotic variable from the EPA dataset along with the Wilks' Lambda *P*. The variables with the highest correlation with discriminant function (in bold) contribute most to the group separation (lakes inhabited by *D. parvula *or *D. retrocurva*).

Of the 97 lakes analyzed from the Department of Natural Resources (DNR) in Wisconsin, there were 16 lakes with only *D. parvula*, 79 lakes with only *D. retrocurva*, and two lakes with both species. In both co-occurrence lakes, one species dominated while the other was rare or only present in a single sampling period throughout the year.

**Figure 3:**
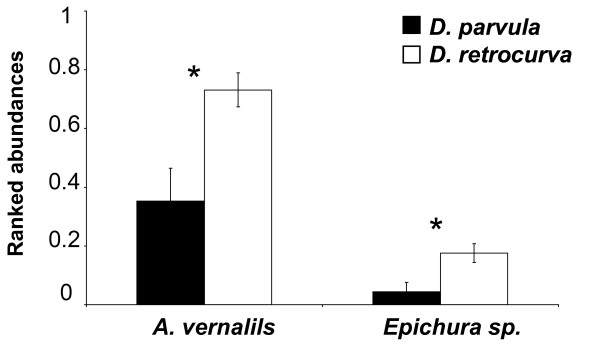
**Invertebrate predators that differed across lakes inhabited by *D. parvula *or *D. retrocurva***. Means (± 1 SE) for the ranked abundances (log transformed) of the invertebrate predators, *A. vernalis *and *Epischura *sp. for the DNR dataset. Univariate ANOVA's were run to test for difference between means in habitats with either *D. parvula *and *D. retrocurva *(* = p < 0.001).

The DA indicated that invertebrate predators significantly discriminated between lakes inhabited by *D. parvula *and those inhabited by *D. retrocurva *(Table 2). The abundance of two copepod predators, *Acanothocyclops vernalis *and *Epischura *sp. contributed most to this group separation (Table 2). *Daphnia retrocurva *inhabits lakes with significantly greater abundances of both these invertebrate predators relative to habitats where *D. parvula *resides (Fig. 3).

**Table 2:** Discriminant Analyses on invertebrate predator abundances.

Variable	Canonical variate correlation coefficients
***A. vernalis***	**0.682**
*M. edax*	0.289
*L. kindti*	0.298
*C. punctipennis*	-0.272
*C. flavicans*	0.276
***Epischura sp.***	**0.561**

Wilks' Lambda *P*	0.024

Canonical variate correlation coefficients derived from the discriminant analysis for abundances for each invertebrate predator in the DNR dataset and the Wilk's lambda *P *value. The variables with the highest canonical variate correlations (in bold) contribute most to the group separation (lakes inhabited by *D. parvula *or *D. retrocurva*).

### Laboratory predation experiments

**Figure 4:**
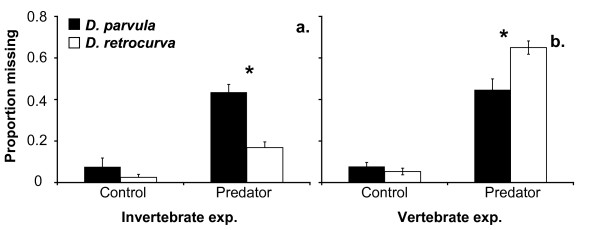
**Relative prey vulnerabilities to tested invertebrate and vertebrate predation**. Mean proportion of prey missing (± 1 SE) for the a) invertebrate predator experiment, and b) vertebrate predator experiment. Asterisks indicate significant differences (p < 0.001) of prey mortality between the prey species used (*D. parvula* or *D. retrocurva*).

For the invertebrate predation experiment, there were significant effects of treatment (control or predator), prey species (*D. parvula *or *D. retrocurva*), and the interaction on proportion of prey missing (Table 3). There was no difference between the proportion of prey missing of the two species in the controls; however, in the predator treatments *D. parvula *suffered significantly higher mortality by *Leptodora kindti *than did *D. retrocurva *(Fig. 4a). The predation rate of *L. kindti *on *D. parvula *was also higher (0.0381 ± 0.01) than on *D. retrocurva *(0.01252 ± 0.002), (mean ± SE).

**Table 3:** Prey vulnerabilities to tested invertebrate and vertebrate predation.

Invertebrate Predation Experiment				
Source	*df*	MS	F	*P*
treatment	1, 46	2.880	65.994	< 0.001
species	1, 46	0.234	5.362	0.025
treatment*species	1, 46	0.215	4.928	0.031

**Vertebrate Predation Experiment**				

treatment	1, 28	2.682	147.589	< 0.001
species	1, 28	0.060	3.322	0.079
treatment*species	1, 28	0.114	6.287	0.031

Results for ANOVA's on the proportion missing prey following the invertebrate and vertebrate predation experiments.

In the vertebrate predation experiment, there were significant effects of treatment (control vs. predator) and treatment by species interaction for the proportion on prey missing (Table 3). There was no significant difference between the proportion of prey missing for *D. parvula *and *D. retrocurva *in the control treatments, but *D. retrocurva *suffered higher mortality by emerald shiners in the predator treatments than did *D. parvula *(Fig. 4b). The predation rate of the fish on *D. retrocurva *was also higher (1.085 ± 0.0296) than that on *D. parvula *(0.638 ± 0.033).

**Figure 5:**
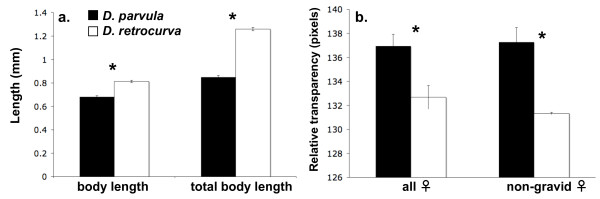
**Morphological measurements on prey populations**. Mean measurements  (± 1 SE) for a) body length and total body length from populations of *D. parvula* and *D. retrocurva* used in the predation experiments, and b) relative transparency from populations used in the vertebrate predation experiment comparing all females and only non-parthenogenic or non-gravid females. Significant pairwise comparisons are indicated with asterisks. (* = p < 0.001).

For all prey populations used in both predator experiments, the ANOVA's indicated *body length*, from top of the compound eye to the base of the carapace (F_1, 98 _= 75.32, P < 0.001) and *total body length*, from highest point of helmet or head to the tip of the tailspine (F_1, 98 _= 394.42, P < 0.001) were both significant by species. *Daphnia parvula *was significantly smaller than *D. retrocurva *and this difference is accentuated when including cyclomorphic features (Fig. 5a). The nested effect of the source population within species (populations used for experiments) was also significant for *body length *(F_2, 97 _= 13.78 P < 0.001) and borderline significant for *total body length *(F_2, 97 _= 3.08 P = 0.0508), indicating that despite much variation across populations, *D. parvula *was consistently smaller than *D. retrocurva*. In addition, *D. parvula *was relatively more transparent than *D. retrocurva *(Fig. 5b), which was still the case when only non-parthenogenic or non-gravid females were analyzed (F_1,48 _= 9.37, P < 0.0036).

### Genetic analyses

**Figure 6:**
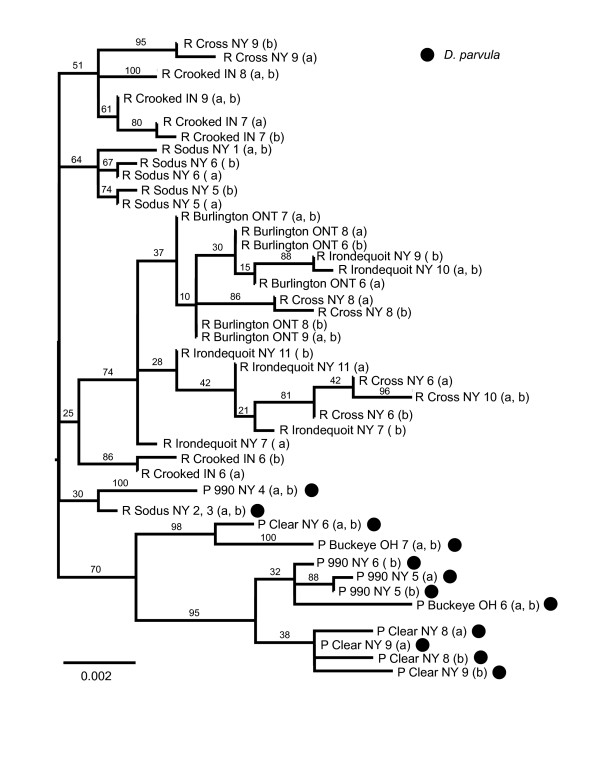
**Phylogenetic analysis including sequences from all four genes**. Phylogenetic relationship (MP tree) of *D. parvula *and *D. retrocurva *populations using the combined sequences of the ND2, HSP90, F6F12 and G6G12 markers. Samples are labelled as "P" for *D. parvula*, "R" for *D. retrocurva*, with all *D. parvula *samples additionally labelled with black circles. Two copies of each individual labelled "a" and "b" were used to represent both alleles at heterozygous sites. The species label is followed by the locality, then state or providence along with the individuals from that population that represent that branch. The numbers above the branches are ML bootstrap support.

**Figure 7:**
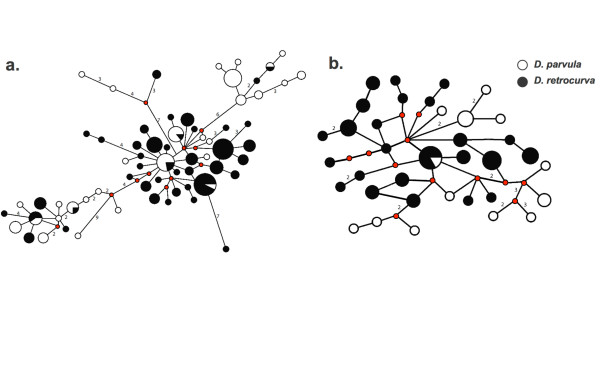
**Haplotype networks for mitochondrial and nuclear sequences**. Median joining haplotype networks for a) mitochondrial (ND2) data, and b) nuclear (HSP90, F6F12, G6G12) data. Each circle represents a unique haplotype and its size is proportional to the number of individuals sharing that specific haplotype. Each small red circle represents hypothetical ancestral haplotypes. Each branch with more than one mutational step is labelled.

The tree was comprised of monophyletic subclades of *D. parvula *and *D. retrocurva *(several with strong support), but there is insufficient information to place the root or to determine if the subclades form reciprocally monophyletic groups (Fig. 6). The genealogical sorting index indicates that the species do form two significantly diverged lineages (Table 4). Mitochondrial and nuclear networks show haplotype sharing but there is differentiation between *D. parvula *and *D. retrocurva *with most of the shared haplotypes having a central or presumed ancestral location (Fig. 7).

**Table 4:** Detection of exclusive ancestry.

Marker	Species	*gsi*	*P*
Mitochondrial	*D. parvula*	0. 6174	< 0.001
(ND2)	*D. retrocurva*	0.7039	< 0.001

Nuclear	*D. parvula*	0.2574	< 0.001
(HSP90, F6F12, G6G12)	*D. retrocurva*	0.3982	0.016

Total	*D. parvula*	0.6614	< 0.001
Evidence	*D. retrocurva*	0.5836	< 0.001

Genealogical sorting index along with the probability statistic testing the null hypothesis that the gene copies labelled *D. retrocurva *and *D. parvula *form a single intermixed group.

**Figure 8:**
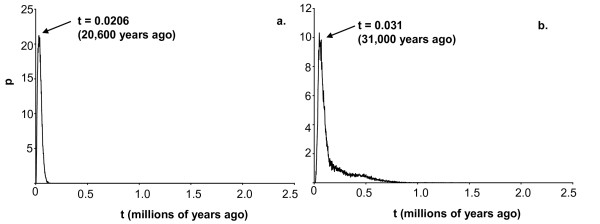
**Estimates of divergence time**. Posterior probabilities of the time of divergence in years of *D. parvula *and *D. retrocurva *when migration priors were set to a) zero migrants/generation, and b) ten migrants/generation.

**Figure 9:**
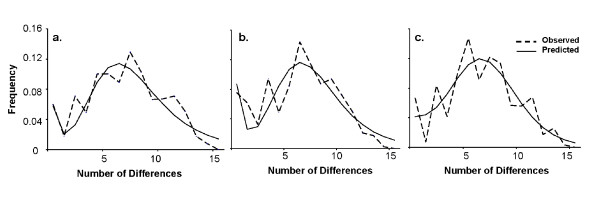
**Evidence for demographic expansion**. Mismatch distribution plot from mitochondrial data for populations of a) both species, b) *D. parvula* and c) *D. retrocurva*. Observed frequencies are dashed lines while predicted frequencies from model are solid lines.

The divergence time (t) was estimated to be at 20,600 years ago when the migration prior was set to zero migrants/generation, and 31,000 years ago with a migration prior set at 10 migrants/generation (Fig. 8). When treating the data as a single group, the mismatch distribution was not significantly different from what is expected under a single expansion (P = 0.74). The τ parameter estimating the date initiating the expansion [70] was 4.765 (95^th ^percentile confidence interval 1.57-11.53; Fig. 9) and the demographic expansion within this group was estimated at 11,405 years ago (95^th ^percentile confidence interval range of 4,000 - 26,000 years ago). The mismatch distributions within *D. parvula *and *D. retrocurva *were not significantly different from the expected distributions under the expansion model (P = 0.78, P = 0.1972, respectively; Fig. 9). The τ parameter estimating the date initiating the expansion was 5.15 (95^th ^percentile confidence interval 1.78 - 12.91) for *D. parvula *and 5.84 (95^th ^percentile confidence interval 2.12 - 4.51) for *D. retrocurva*. Demographic expansion was estimated at 12,331 years ago (95^th ^percentile confidence interval range of 3,000- 31,000 years ago) for *D. parvula *and 13,969 years ago (95^th ^percentile interval range of 5,000 - 35,000) for *D. retrocurva*.

The results of the Analysis of Molecular Variance (AMOVA) of both the individual markers and all four markers combined indicate a relatively small proportion of the total genetic variation is found between the two species. In addition, the fixation indices demonstrate very high differentiation among populations within a species and within populations (Table 5).

**Table 5:** AMOVA results.

Source of Variation	Marker	*df*	Diversity Index	% Variation	*P*
Between species	ND2	1	0.1038	10.38	0.0013
	HSP90	1	0.2498	24.98	0.0373
	All 4 markers	1	0.2542	25.42	0.0079

Among populations within species	ND2	26	0.5946	49.07	< 0.001
	HSP90	11	0.4286	32.15	< 0.001
	All 4 markers	7	0.6434	47.98	< 0.001

Within populations	ND2 HSP90 All 4 markers	133	0.5476	40.54	< 0.001
	HSP90	31	0.5713	42.87	< 0.001
	All 4 markers	55	0.7341	26.59	< 0.001

Hierarchal AMOVA results between species, among populations within species and within populations across both markers.

## Discussion

Our findings illustrate rare co-occurrence of *D. parvula *and *D. retrocurva *in the field with strong spatial segregation in ecologically differentiated lakes supporting ecological isolation. Priority effects and dispersal limitation can contribute to habitat isolation [71,72], but do not predict a pattern of distribution associated with lake type. Moreover, both species appear to be strong dispersers [47,64,73] and unidirectional translocation studies have failed to result in colonization [65]. The rare co-occurrence of *parvula*-like morphotypes with *retrocurva *described by Brooks [42] remains mysterious. We failed to detect *parvula*-like specimens in multiple samples from the same embayment of Lake Ontario examined by Brooks. The rare *parvula*-like morphs in glacial lakes could be the result of recent inflow from shallow adjacent waters or transient ex-ephippial clones of *parvula.*

What are the ecological traits associated with isolation? Kerfoot and Weider [63] have elegantly shown that evolution of the helmet size (defensive structures that are the main morphological difference between *D. retrocurva *and *D. parvula*) can be attributed to historical changes in invertebrate predation. Here we show that this process may also contribute to the spatial segregation of these two species in nature, as *D. retrocurva *inhabit larger lakes that contain higher abundances of invertebrate predators relative to lakes where *D. parvula *is found. Although the data collected did not permit us to examine if fish, or vertebrate predators discriminated between the habitats encountered by these two species, the abundance of *D. parvula *has been found to be positively associated with the abundance of planktivorous fish [64]. We also were unable to make a direct association between the abiotic and biotic factors compared in this study since these data were obtained from different sources, yet deeper aquatic habitats can have higher abundances of invertebrate predators of *Daphnia *because depth affords a refuge from visual predators for conspicuous invertebrate predators [74]. Unexpectedly, the lake data failed to reveal an association between the abundance of *Leptodora *and *Daphnia *species. One possible explanation for the lack of association is the difficulty in quantifying abundances of vertically migrating *Leptodora *in daylight hauls [75-77], yet the abundances of *D. retrocurva *and *Leptodora *have been found to be positively associated in other studies [48].

We also found that *D. parvula *has an advantage relative to *D. retrocurva *in the presence of visual, vertebrate predation which may be a consequence of the smaller body size and greater transparency of *D. parvula *(Fig. 5). Additionally, *D. retrocurva *has an advantage over *D. parvula *in the lab to the invertebrate predator tested. Thus, the present study and existing translocation and longitudinal evidence suggest that *D. parvula *and *D. retrocurva *have a selective advantage to different habitat-associated predators. It is possible that predation may promote "immigrant inviability" between these two species, or selection against migrants between locally adapted populations, which is a direct result of natural selection reducing gene flow [78,79]. The differing predation regimes likely contribute to the spatial segregation of these sister species, but additional local processes may to contribute as well in this system [65]. Our study found that abiotic chemical variables (i.e. nutrients, conductivity) did not discriminate between the two species' habitats, yet other processes that were not included in this study could also contribute to their habitat segregation such as competition, the interactive effects of multiple predators, priority effects, and dispersal [80-83]. Further studies are needed to evaluate the role these other factors may play in facilitating habitat isolation between *D. parvula *and *D. retrocurva*, yet evidence suggests that predation is a key factor in determining zooplankton community composition and can reduce the importance of other factors influencing the establishment of *Daphnia *populations [81,84].

Our results support postglacial genetic divergence between *D. parvula *and *D. retrocurva *despite a mixed breeding system and high dispersal. The networks, single demographic expansion patterns, and divergence estimates based on nuclear and mitochondrial DNA are consistent with intermediate divergence and postglacial isolation. This postglacial timing of divergence is consistent with the origins of the deep temperate glacial lakes [85,86] that constitute the main habitat of *D. retrocurva*. We note that the findings of intermediate divergence do not rule out actual or potential gene flow between *D. retrocurva *and *D. parvula*. The AMOVA reveals a relatively low proportion of the total genetic variation is found between the two species and genetic divergence associated with ecological processes is often characterized by intermediate divergence with ongoing gene flow [87].

Although *Daphnia *are known to have high dispersal and colonization rates [21], the fixation indices suggest strong genetic differentiation among intraspecific populations (Table 5), suggesting natural selection may also be operating within species as well. Therefore, selection against migrants may lead to a reduction in gene flow in this system on two scales, between two species and the two larger classes of habitat types they reside in, and within a species and the individual local habitat each population resides in. According to island population models, as long as the island population size increases more rapidly than the number of migrants, the per-generation genetic contribution of migrants should decrease and an equilibrium is reached [72] which is likely in *Daphnia*. Their cyclic parthenogenic reproduction, rapid population growth, and priority effects supported by a large resting bank coupled with local adaptations may allow residents to maintain large populations relative to incoming migrants. Many studies illustrate strong, local adaptations in *Daphnia *populations and the reduced success of invaders [21,72,81], this high genetic differentiation among intraspecific *Daphnia *populations often leads to a monopolization effect [67,72,88,89] in which predation, competition, reproduction, ontogeny, and tolerance to eutrophication have been implicated to promote [72,88-90].

Conflicting models have been proposed whereby mixed breeding systems either slow [12] or accelerate divergence [18,23,26]. In our study, despite their young age, *D. retrocurva *and *D. parvula *illustrate ecological divergence with respect to their habitats, morphology, and predator vulnerabilities suggesting possible selective advantages to each in their respective environments. Both species typically have a mixed breeding system with relatively infrequent sex [42,44,45,47] and illustrate strong dispersal capabilities [47,64,73]. Although some believe that populations with mixed breeding systems are less likely to yield discrete or distinct groups relative to populations with strictly sexual or asexual reproduction [12], this study illustrates two groups that have diverged into two distinct lineages and maintained this divergence with a mixed breeding system coupled with high dispersal, and more importantly over a relatively short period of time. Lynch and Gabriel [89] propose that a single generation of sex can reveal 50-75% of the genetic variance hidden by asexual reproduction. Therefore, reproduction marked by cyclic parthenogenesis could allow such rapid divergence through the combined benefits of rapid population growth rates that asexual reproduction offers along with the release of genetic variation in the few episodes of sexual reproduction [91]. We postulate this mixed breeding system permits such rapid postglacial divergence of *D. parvula *and *D. retrocurva*, with selection against migrants (immigrant inviability) maintaining this divergence by reducing the homogenizing effects of dispersal.

## Conclusions

We present evidence for rapid ecological, morphological, and genetic divergence of sister species of lacustrine cyclic parthenogens. The species differ in genetically-based defensive structures and predation regimes. Predation has been implicated as a selective force promoting divergence among taxa in only a few animal lineages. The present study suggests a role for predation in facilitating habitat isolation in young sister species with a mixed breeding system. However, multifarious selection can bring about reproductive isolation more effectively than selection from a single factor, and the habitats of *D. retrocurva *and *D. parvula *differ in numerous ways that could affect selection. Although predation has been implicated as a key factor in determining community composition and establishment of *Daphnia *[81,84], other factors need to be further evaluated. Our results do indicate that a better understanding of the evolution of the numerous presumed intraspecific defensive-morphotypes of cladocerans is needed. Intermediate divergence of aquatic cyclic parthenogens is likely more common than previously thought.

## Methods

### Habitat differentiation

We obtained occurrence data from the United States Environmental Protection Agency's (EPA) Environmental Monitoring and Association Proposal (EMAP), http://www.epa.gov/emap/. These data were collected from 64 lakes and ponds in the northeastern United States in the months of July through September from 1991 to 1994. From this study, data were extracted on the presence or absence of *D. parvula *or *D. retrocurva *within each habitat, along with physical and chemical measurements of: ionic strength (M), pH, total nitrogen (μg/L), total phosphorus (μg/L), trichomatic chlorophyll A (μg/L), total suspended solids (μg/L), secchi depth (m), calculated alkalinity (meq/L), turbidity (NTU), average depth of lake (m), lake volume (m^3^), lake surface area (ha), and % of watershed in human disturbed land. The methods used to measure each variable are described at http://www.epa.gov/emap/. We coded the presence/absence data in each site for both *D. parvula *and *D. retrocurva*. There was one lake in the EPA abiotic dataset that included both species, and in this lake *D. parvula *was rare. Data from this co-occurrence lake were removed from the analysis (see below) due to the small sample size.

Separate data collected by the Wisconsin Department of Natural Resources (DNR) [92] from 1973-1974 from 99 lakes throughout Wisconsin were used to test for differences in invertebrate predator assemblages across the species' habitats. We were unable to assess differences between vertebrate predator assemblages (fish) due to incomplete or missing data. Each lake was sampled four times that year (between the months of April through November) where zooplankton and invertebrate predator abundances were recorded. These data include ranked abundances of the following invertebrate predators: the copepods *Acanthocyclops vernalis*, *Epischura sp.*, and *Mesocyclops edax*; the larval dipterans *Chaoborus flavicans*, *C. punctipennis*, and *Chaoborus sp*.; and the cladoceran *Leptodora kindti*. The abundance data were ranked according to the absolute abundances found in the field as follows: 0 = absent, 1 = rare (3 or less), 2 = uncommon (4-50), 3 = common (51-1,000), and 4 = abundant (> 1000). We coded the presence/absence of *D. parvula *and *D. retrocurva*. Since the timing of the four periods sampled varied across lakes, the mean values for each lake were used in the analyses. This dataset had two lakes where the species co-occurred (never during the same time period), which were removed from the analysis (see below) due to the small sample size.

Because abiotic and biotic variables are often correlated [28], the datasets were analyzed with a Discriminant Analysis (DA) in SPSS (ver. 11.5, 2006). Two separate DA's were run, one including the abiotic data (EPA) and the other including the biotic data (DNR). Some data were transformed to meet the assumption of normality. We tested whether the variables in a data set (abiotic or biotic) yielded significant discrimination between lakes inhabited by *D. parvula *versus *D. retrocurva *and the correlation of these variables with the discriminant function was used to determine the relative contribution of each variable to the group separation. In the abiotic DA we retrieved three variables that significantly discriminated between lakes inhabited by *D. parvula *or *D. retrocurva *(lake volume, area, and depth). Since all three variables were mutually correlated, we ran an Analysis of Variance (ANOVA) on lake volume since it had the highest correlation with the discriminant function, with species as the fixed effect. For the invertebrate predators, an ANOVA was used to test for differences between individual predator means indicated by the DA that best discriminate between habitats with *D. parvula *or *D. retrocurva*.

### Laboratory predation experiments

We tested whether *D. parvula *and *D. retrocurva *differ in their vulnerabilities to habitat-specific predators and if they exhibit morphological traits that are known to affect vulnerabilities towards the respective predators. One experiment used an invertebrate predator (*Leptodora kindti*) while the other a vertebrate predator (emerald shiners, *Notropis atherinoides*). In addition, morphological measurements were taken of relative body size and transparency for populations used in the predation experiments.

During late summer (August and September) we collected *D. parvula *from Buffalo, NY (43° 01' 39.37'' N, 79° 48' 72.39'' W) and *D. retrocurva *from embayments of western Lake Ontario (43° 11' 22.04'' N, 77° 31' 75.09'' W from Irondequoit Bay, NY in August and 43° 17' 59.83'' N, 79° 48' 16.81'' W from Burlington, Ontario in September). *Daphnia *was reared for two weeks prior to the experiment at constant densities in 50-ml vials filled with synthetic lake water (96 mg NaHCO_3_, 60 mg CaSO_4 _2H_2_O, 60 mg MgSO_4 _and 4 mg KCL in 1 L of double distilled water; EPA 2002) with 10 *Daphnia *per vial at 21°C with a 24 h light photoperiod. All vials were treated with cetyl alcohol (CH_3 _(CH_2_)_15_OH) to prevent the *Daphnia *from getting caught in the surface tension. Every three days, 1000 μl of *Selenastrum capricornutum *suspensions were added to each vial of *Daphnia *as a food source.

*Leptodora kindti *was field-collected from Lake Ontario (43° 11' 22.04'' N, 77° 31' 75.09'' W), one week prior to the experiment. *Leptodora *were individually maintained in 50 ml vials filled with synthetic freshwater at 21°C with 24 h light photoperiod. Every three days *L. kindti *was given zooplankton collected from the field as a food source.

Emerald shiners (from 50 to 64 mm long in standard length) were obtained from a bait shop and were previously field-collected from the Niagara River, Buffalo, NY. Although the source of these predators were from the Niagara River, emerald shiners are often the dominant planktivorous fish in lakes with *Daphnia *representing one of their main food sources [93,94]. Prior to the experiment, the fish were kept in 10 L tanks with treated tap water at room temperature (21°C) and fed a mix of freshwater fish food (Tetrafin) and zooplankton collected from the field. Both invertebrate and vertebrate predators were starved for 24 hours prior to the experiments.

For the invertebrate predator experiment, all experimental trials took place in 90 ml plastic cups with 50 ml of synthetic fresh water treated with cetyl alcohol 24 hours prior to the experiment. On the day of the experiment, 20 adult female *D. parvula *(Buffalo population collected in August) or *D. retrocurva *(Irondequoit Bay population) were added to a cup, which was then randomly assigned 1 or 0 adult *L. kindti *(predator and control, respectively). There were 20 replicates of each prey treatment within the predator treatment and 4 replicates of each prey treatment within the control treatment. After 24 hours (21°C, 24 h light photoperiod) the predator was removed and surviving prey were counted.

For the vertebrate predator experiment, all experimental trials took place in 3.8 L plastic containers with 3 L of synthetic lake water at room temperature (21°C). On the day of the experiment, 50 adult females of either *D. parvula *(Buffalo population collected in September) or *D. retrocurva *(Burlington population) were added to a container that was randomly assigned 1 or 0 fish (predator and control, respectively). There were 3-4 replicates of the predator treatments and 2 replicates of the control treatments for both prey species on each of 3 days (total: predator 10 replicates, 6 control replicates, for each prey species). The number of replicates in the vertebrate predator experiment was limited by the number of available *Daphnia *and each replicate used a novel predator. After one hour the predators were removed and the surviving prey were counted.

Prey mortality was equated with the proportion of prey missing. The proportion of prey missing was arcsine square root transformed and analyzed by factorial ANOVA with predator, prey, and the interaction as fixed effects. Post hoc tests with Tukey correction were performed to detect any pairwise differences among mean proportion prey missing between species.

The predation rate coefficient *K *was calculated in the predator treatments only. We calculated the predation rate coefficient *K *derived from the Lotka and Volterra equation

where *P *is the number of prey per liter and *X *is the number of predators per liter. The coefficient *K *was then calculated by the equation

where *P*_I _is the initial concentration of prey per experimental unit, *P*_T _is the concentration of prey per experimental unit at the end of the trial, *X *is the number of predators per experimental unit, and *T *is the duration (in hours) of the trial [95]. Although this equation does not take into consideration reproduction or intrinsic mortality of both prey and predation, in our experiments no reproduction was observed and prey mortality was quantified by the number of prey missing rather than number of dead prey. Although we observed a small proportion of prey missing in the controls treatments, this random effect was likely constant among both control and predator treatment as the higher proportion of prey missing was consistent in the predator treatment replicates.

Following the experiments, morphological measurements were taken on 25 adult females of *D. parvula *and *D. retrocurva *for each predation experiment (50 total for each species) that were randomly sampled from the laboratory source populations that provided the *Daphnia *for each predation experiment (individuals from the populations not used in the experiments). From populations from both predation experiments, we measured the *body length *(from the top of the compound eye to the base of the carapace), and the *total body length *(from the highest point of the helmet or head to the tip of the tail spine). Measurements were taken with a dissecting microscope and ImageJ (ver. 1.37, 2006). Since *D. parvula *cannot produce a helmet, these two measurements were used to quantify the body size of *D. parvula *relative to that of *D. retrocurva*, with and without the cyclomorphic features exhibited (helmets and tail spines). We ran separate nested ANOVA's to test for differences in body length and total body length with species as the fixed effect (*D. parvula *or *D. retrocurva*), and source population nested within species (locality and time collected for either for the invertebrate or vertebrate predation experiment) as a random effect. Both variables were log transformed to meet the assumptions of normality and homogeneity of variances, although raw means are illustrated in the figures.

From populations of *D. parvula *and *D. retrocurva *used for the vertebrate predation experiment, we additionally quantified the relative transparency using a dissecting microscope and the "Histogram" analysis of ImageJ. Each individual *Daphnia *was photographed in grey scale under identical magnification and conditions, using only transmitted light. The relative transparency was quantified from the mean pixel intensity (0 to 255 for pixel shades from black to white) for the specimen image. Following confirmation that the photo conditions were standardized across both species, an ANOVA was run to test for differences in transparency between *D. parvula *and *D. retrocurva*. An identical analysis was also run on transparency only among non-gravid females from both species, as the presence of parthenogenic eggs can affect detection of prey by vertebrate predators [96]. All dependent variables were log transformed to meet the assumptions of normality and homogeneity of variances, yet raw means are illustrated in the figures.

### Genetic analyses

Specimens of *D. parvula *and *D. retrocurva *were collected from populations (n = 29) throughout the continental United States and Canada (see Additional file 1, Table S1). Most populations were from areas in Northern United States and southern Canada where the two species' distributions overlap (Fig. 2, Additional file 1: Table S1). We obtained from 1-10 individuals per population (average 5.44 individuals/population).

Total genomic DNA was extracted using Quick Extract (Epicentre). Samples were homogenized in 35-45 μl of Quick Extract solution, then incubated at 65°C for 2 h and 98°C for 10 minutes, and stored at -20°C. One mitochondrial and three nuclear markers were sequenced from the extracted DNA. A 631 bp fragment of mitochondrial protein-coding NADH-2 (ND2) was amplified with the primers (5' - GTTCATGCCCCATTTATAGGTTA - 3') and (5'- GAAGGTTTTTAGTTTAGTTAACTTAAAATTCT-3'). The primers (5'-TTACGAGTCCAGATGGGCTT-3') and (5'- ATCCGTTATGAATCCCTGACTGA-3') were used to amplify a 669 bp fragment of protein-coding HSP90 gene. A 433 bp fragment of the nuclear rab GTPase (F6F12) gene was amplified with the primers (5'- CGTTTCGAATTGGCTTACTGA-3') and (5'- CATCGTTATCTGTCTACGTCTTGAA-3'), while a 534 bp fragment of the translation initiation factor (G6G12) gene was amplified with the primers (5'- AGAAATTCAACATGCCCAAGA-3') and (5'- CGTCGACGAAGTTGACAGTAT-3') [97]. Each Polymerase Chain Reaction (PCR) was 50-54 μl in total and consisted of 4-8 μl of extracted DNA, 5 μl of10 × PCR buffer [50 mM KCl, 1.5 mg MgCl_2, _10 mM Tris-HCl pH 8.3, 0.01% (w/v) gelatin], 1.5 μl of DNTP's (2 mM of each), 1 μl of 10 μM of each primer and 1 μl of standard Taq DNA polymerase. Each PCR was conducted on a MJ Thermocycler with the following conditions for the mitochondrial (ND2) gene: 40 cycles at 94°C for 30 s, 48°C for 30 s, and 72°C for 1 min, and a final extension at 72°C for 7 min. The PCR temperature profiles for the nuclear (HSP90) gene were 40 cycles at 94°C for 30 s, 50°C for 30 s and 72°C for 1 min. with a final extension at 72°C for 5 min. For the nuclear (F6F12) gene the PCR temperature profile was 40 cycles at 94°C for 30 s, 58°C for 30 s and 72°C for 1 min. with a final extension at 72°C for 5 min, identical temperature profiles were used for the nuclear (G6G12) gene with the exception of the annealing temperature set at 53°C rather than 58°C. Sequences of all nuclear and mitochondrial PCR products were obtained in both directions by Genaissance Pharmaceuticals or the University of Washington and were both assembled and edited with SEQUENCHER ver. 4.2 (Gene Code) then manually aligned in SE-AL 2.0 [98].

An individual was considered heterozygous when overlapping peaks (lower peak > 95% of higher peak) were observed at any given site on the sequence electropherogram. Individuals with multiple heterozygous sites were cloned with the Invitrogen TOPO TA kit to determine the different alleles at each site with observed heterozygosity. The QIAprep Spin Miniprep Kit (QIAGEN) was used for plasmid purification and the respective primers for each particular locus were used to sequence the cloned inserts. For each individual, six cloned fragments were sequenced to help detect cloning artifacts [99]. The combined data for all four makers was a 2268 bp sequence with both alleles represented for each individual for the nuclear genes. All individuals sequenced for the F6F12 and G6G12 markers were also represented with sequences for the HSP90 and ND2 markers. DNA quality appeared to prohibit sequencing of all genes for all individuals. Therefore, we conducted analyses for 1) individuals represented by sequences for only the mitochondrial ND2 gene, 2) individuals represented by sequences with only the three nuclear genes, and 3) individuals represented by sequences for all four genes (total evidence).

The number of individuals sequenced from each marker used in the analyses can be seen in Additional file 1: Table S1. Each individual was represented by two copies of each sequence to account for both alleles at a heterozygote site in the nuclear markers, while the mitochondrial (ND2) gene was replicated twice to correspond with the two copies of each nuclear gene sequence for the analyses. We performed a phylogenetic analysis on the total evidence data set (all four genes combined) using Maximum Likelihood (ML) method with RAxML [100] and a file to partition the alignment by codon position and gene. For all analyses a GTR substitution model was implemented for the analysis and unique model parameters were optimized and applied for each partition. Support values were estimated using 100 bootstrap replicates.

Because the transition from polyphyly to monophyly is continuous, a categorical description of divergence can fail to identify significant divergence before monophyly is reached [101]. Thus, detecting divergence in closely related or recently diverged species phylogenetically can be difficult if monophyly has not been reached. The recently described Genealogical Sorting Index (*gsi*) allows one to detect significant genealogical divergence before monophyly by quantifying the relative degree of exclusive ancestry of a labelled group on a rooted tree topology. Since branch tips along a tree generally represent gene copies of a given locus, the value of *gsi *is the degree of genealogical exclusivity among the sampled gene copies. The calculated *gsi *value ranges from 0-1, with 0 representing panmixia and 1 representing monophyly and permutation tests provide a statistical test of significance for this value [101].

We calculated the *gsi *value for each species (*D. parvula *and *D. retrocurva*) and tested the null hypothesis that the gene copies labelled *D. retrocurva *and *D. parvula *form a single intermixed group. Because strongly unbalanced group representation can result in a decreased power to detect significant exclusive relationships [101] we determined the *gsi *for the mitochondrial data, the nuclear data, and the total evidence data after randomly removing *D. retrocurva *individuals from the data to achieve a balanced sampling scheme, since this species was over represented compared to *D. parvula*.

A median-joining haplotype network was constructed for the mitochondrial data and the three combined nuclear gene sequence data using NETWORK ver. 4.5.0.0 [102]. In order to further estimate divergence time we used the isolation with migration- analytic (IMa) model, which was designed to analyze recently separated populations (or in this case, species) not at equilibrium. This model estimates six demographic parameters including gene flow rates and time of divergence while generating relative likelihoods/posterior probabilities [103]. Unfortunately we could not get reliable migration or gene flow estimates in IMa likely due to the increased number of parameters in the model (J. Hey communication); therefore, we used IMa solely for estimating divergence time. Since the degree of current gene flow between *D. parvula *and *D. retrocurva *is unknown we ran two IMa analyses, one with the migration prior set to zero migrants per generation and another with ten migrants per generation between the two species which we treated as populations. For both analyses, we used the Hasegawa-Kishino-Yano model [104] and the Infinite Sites mutation model for the mitochondrial and nuclear markers, respectively. The mutation rates entered in the model were the number of bp in the particular marker multiplied by 3.3 × 10^-6 ^mutations/site/year for the mitochondrial marker and 1.05 × 10^-7 ^mutations/site/year for the nuclear markers [105], assuming 5 generations per year; previous estimates adjusted to seasonal duration of localities [106,107]. The analysis included 30 million generations post burn-in (1 million generations) with six Metropolis-coupled Markov chains with the first heating parameter for the linear heating scheme set to 0.05. Tajima's D test and the four-gamete test (DNASP ver. 4.0) indicate all markers are consistent with the expectations of neutrality and the nuclear markers show no evidence of recombination, both assumptions of the IMa model.

Sequences from the rapidly evolving mitochondrial ND2 gene were used to determine the demographic history of both *D. parvula *and *D. retrocurva *lineages. Three mismatch distributions were calculated from the number of differences between pairs of haplotypes within and between species in ARLEQUIN [108]. The Sum of Square Deviations (SSD) was computed for each species separately then tested whether the observed distributions deviated significantly from those expected under the population expansion model using 10000 permutation replicates. We used the parameter τ to estimate the time since the expansion (t) using the equation t = τ/2 u, where u is the mutation rate per sequence per generation [109] assuming a mutation rate of 6.6 × 10^-8^/site/generation [110] and five generations/year.

We ran hierarchical Analysis of Molecular Variance (AMOVA) in ARLEQUIN [108] to determine the total genetic variation explained by differences *(i) *between the two species *D. parvula *and *D. retrocurva*, (F_CT_), *(ii) *among populations within a species, (F_SC_), and *(iii) *within a population (F_IS_).

## Authors' contributions

KSC and DJT designed the study. KSC performed laboratory experiments, molecular lab work and performed all the analyses. KSC and DJT were both involved in analysis interpretation and manuscript composition. All authors read and approved the final manuscript.

## Supplementary Material

Additional file 1 **Appendix Table S1 - List of populations used in genetic analyses**. Click here for file
