# Supplementary material for: Rapid ecological isolation and intermediate genetic divergence in lacustrine cyclic parthenogens
Source: BMC Evol Biol. 2010 Jun 5;10:166. doi: 10.1186/1471-2148-10-166 (PMC2895610; doi:10.1186/1471-2148-10-166)
Supplement: Additional file 1 — Appendix Table S1 - List of populations used in genetic analyses. [file 1471-2148-10-166-S1.DOC]

**Appendix Table S1 – List of populations and samples used in genetic analyses.**

List of localities and sample sizes used for each species for all phylogenetic and population genetic analyses when considering only the mitochondrial ND2 marker, only the nuclear HSP90 marker, and all four markers combined.

| **Species** | **Site** | **Location** | **Latitude** | **Longitude** | **N**  **ND2** | **N**  **HSP90** | **N**  **all markers** |
| --- | --- | --- | --- | --- | --- | --- | --- |
| *Daphnia retrocurva* | Bantam Lake | CT, U.S.A. | 41.702854 | -73.219641 | 4 | - | - |
| *D. retrocurva* | Big Barbee Lake | IN, U.S.A. | 41.284420 | -85.703571 | 5 | 5 | - |
| *D. retrocurva* | Crooked Lake | IN, U.S.A. | 41.256976 | -85.479839 | 9 | 7 | 4 |
| *D. retrocurva* | Kuhn Lake | IN, U.S.A. | 41.284640 | -85.693300 | 5 | - | - |
| *D. retrocurva* | Tippicanoe Lake | IN, U.S.A. | 41.326944 | -85.759498 | 5 | 4 | - |
| *D. retrocurva* | Waubee Lake | IN, U.S.A. | 41.387548 | -85.830159 | 5 | - | - |
| *D. retrocurva* | Fine Lake | MI, U.S.A. | 42.442873 | -85.295495 | 4 | - | - |
| *D. retrocurva* | Maple Lake | MI, U.S.A. | 47.641857 | -93.719725 | 4 | - | - |
| *D. retrocurva* | Sherman Lake | MI, U.S.A. | 42.349646 | -85.390019 | 5 | - | - |
| *D. retrocurva* | Teeple Lake | MI, U.S.A. | 42.628231 | -83.550404 | 5 | - | - |
| *D. retrocurva* | Prairie Lake | MN, U.S.A. | 47.309361 | -93.538823 | 5 | - | - |
| *D. retrocurva* | Splithead Lake | MN, U.S.A. | 47.062387 | -93.485860 | 4 | - | - |
| *D. retrocurva* | Cross Lake | NY, U.S.A. | 43.112599 | -76.480942 | 10 | 7 | 4 |
| *D. retrocurva* | Irondequoit Bay | NY, U.S.A | 43.186779 | -77.527335 | 11 | - | 4 |
| *D. retrocurva* | Big Hill Lake | WI,, U.S.A. | 44.157370 | -89.160090 | 4 | 2 | - |
| *D. retrocurva* | Lake Mendota | WI,, U.S.A. | 43.095952 | -89.372325 | 5 | 2 | - |
| *D. retrocurva* | Burlington Bay | ONT, Canada | 43.950000 | -79.433340 | 10 | 5 | 5 |
| *D. retrocurva* | St. George Lake | ONT, Canada | 43.948848 | -79.435955 | 2 | - | - |
| *D. retrocurva* | Sodus Point | NY, USA | 43.026635 | -78.77934 | 5 | 4 | 5 |
| *Daphnia parvula* | Callion Lake | AR, U.S.A. | 33.327560 | -92.535882 | 3 | - | - |
| *D. parvula* | Paris Lake | AR, U.S.A. | 25.271673 | -93.723320 | 2 | - | - |
| *D. parvula* | Sequoia Lake | CA, U.S.A. | 36.731767 | -118.994185 | 3 | 3 | - |
| *D. parvula* | Wellington | KA, U.S.A. | 37.213506 | -97.528537 | 6 | - | - |
| *D. parvula* | Buckeye Lake | OH, U.S.A. | 39.923939 | -82.468558 | 7 | 7 | 2 |
| *D. parvula* | Clear Lake | NY, U.S.A. | 42.552806 | -78.853205 | 10 | 7 | 3 |
| *D. parvula* | Buffalo | NY, U.S.A. | 43.027462 | -78.710806 | 4 | 3 |  |
| *D. parvula* | Amerhst990 | NY, U.S.A. | 43.027210 | -78.710883 | 9 | 6 | 2 |
| *D. parvula* | Farmers Pond | ONT, Canada | 43.520487 | -80.567185 | 2 | - | - |
| *D. parvula* | Toronto Pond | ONT, Canada | 43.641229 | -79.466742 | 5 | - | - |
